# A commentary on ‘Does speed equal quality? Time pressure impairs minimally invasive surgical skills in a prospective crossover trial’ (Int J Surg 104 (2022) 106813)

**DOI:** 10.1097/JS9.0000000000000318

**Published:** 2023-03-24

**Authors:** Ziwei Li, Yan Shu

**Affiliations:** a Central Laboratory; b Department of Clinical Laboratory, Chongqing University FuLing Hospital, Chongqing, People’s Republic of China

*Dear Editor*,

Time pressure is an important influencing factor in clinical work. Even though less operation time reduces patient exposure to surgical stressors or anesthetic time, leading to less infection and bleeding, lots of research indicate management failure of time pressure may lead to operating error and nosocomial damage^1^. A better understanding of the effect of time pressure in the clinic, especially during surgery, is meaningful work.

In the impressive study from the University Hospital Carl Gustav Carus, the author stated that time pressure during minimally invasive surgery might improve procedural time but impair the quality of surgical performance in terms of the incidence of errors and force exertion. Accumulation of experience seems to be the only solution. The study makes time pressure detectable and indicates the antipressure ability of surgeons mainly based on experience accumulation.

However, some issues in this article may need further interpretation. First, before the test, the surgeon group did not get the same training or pretest as the novice group. Although surgeons have more experience in daily surgery, the same training is still needed to diminish the difference in skills in four tasks: PEG (percutaneous endoscopic gastrostomy) transfer, precision cutting, balloon resection, and surgical sutures and knots. For example, in this research, the novice group spend much less time than the surgeon group only in PEG transfer but much more time in the other three tasks. And actually, the surgeon group showed higher error occurrences than the novice group in all kinds of error statistics except balloon perforation. It is hard to believe surgeons are less skillful. Lack of training can be a proper explanation for such a paradox. And interestingly, no matter whether time stress is applied or not, the surgeon group showed a noteworthy lower stress level than the novice group even though they had more errors and spent a long time during testing. When compared with invoices, the self-cognition of the surgeon may have a role that cannot be ignored in stress management^2^.

Second, the difference between novice surgeons and skillful surgeons is not only in experience but also differs greatly in standardization of operation and operational fluency. Indeed, excessive force can lead to a medical injury. But in this research, the force of both groups is all far from 10.3N^3^, which leads to human small intestine perforations. On the other side, weak or fluctuating forces may also be related to a lack of self-confidence or hesitation, which leads to a stumbling surgery process. The meaning of force diversity should be explained not only in static but also in dynamic. Adding action mode analysis to force recording may help us have better awareness of the effect of time pressure.

Finally, before and after the application of pressure, the experience of operating under no pressure can be ignored for the novice who has been trained for a long time, but it can make a big difference for the surgeons who have not received pretest and only rely on surgical techniques. As surgeons themselves have good operational skills, only one time of test could help them get a better understanding and movement to achieve better performance in the pressure-applied task.

In conclusion, dealing with time pressure is an important issue during minimally invasive surgery. Distler provides a measurable method for stress evaluation and reveals that accumulation of experience is important for time pressure management. It is crucial that not only static force measurement but also dynamic recording and analysis should be considered. And with a larger sample size, the differences between surgeons with different seniority may better illustrate the role of time pressure in surgery.

## Ethical approval

This manuscript was based on the previously published study; thus, no ethical approval is required.

## Patient consent

N/A

## Sources of funding

Chongqing Medical Scientific Research Project (Joint project of Chongqing Health Commission and Science and Technology Bureau) (2020FYYX189).

## Author contribution

Z.L.: drafted the article; Y.S.: reviewed the article.

## Conflicts of interest disclosure

There are no conflicts of interest.

## Research registration unique identifying number (UIN)

None.

## Guarantor

Yan Shu, Department of Clinical Laboratory, Chongqing University FuLing Hospital, Chongqing 408000, People’s Republic of China, E-mail: 635458283@qq.com

## Data availability statement

Data sharing is not applicable to this article as no new data were created or analyzed in this study.

## Provenance and peer review

None.
